# Effects of Aging on Osteosynthesis at Bone–Implant Interfaces

**DOI:** 10.3390/biom14010052

**Published:** 2023-12-30

**Authors:** Alexa K. Pius, Masakazu Toya, Qi Gao, Max L. Lee, Yasemin Sude Ergul, Simon Kwoon-Ho Chow, Stuart Barry Goodman

**Affiliations:** 1 Department of Orthopaedic Surgery, School of Medicine, Stanford University, Stanford, CA 94063, USA; apius@stanford.edu (A.K.P.); mtoya@stanford.edu (M.T.); qigao7@stanford.edu (Q.G.); maxlee12@stanford.edu (M.L.L.); yaseminsudeergul@gmail.com (Y.S.E.); skhchow@stanford.edu (S.K.-H.C.); 2 Department of Bioengineering, Stanford University, Stanford, CA 94305, USA

**Keywords:** osteosynthesis, bone regeneration, aging, inflammatory response, OI

## Abstract

Joint replacement is a common surgery and is predominantly utilized for treatment of osteoarthritis in the aging population. The longevity of many of these implants depends on bony ingrowth. Here, we provide an overview of current techniques in osteogenesis (inducing bone growth onto an implant), which is affected by aging and inflammation. In this review we cover the biologic underpinnings of these processes as well as the clinical applications. Overall, aging has a significant effect at the cellular and macroscopic level that impacts osteosynthesis at bone-metal interfaces after joint arthroplasty; potential solutions include targeting prolonged inflammation, preventing microbial adhesion, and enhancing osteoinductive and osteoconductive properties.

## 1. Introduction

Total joint replacement is one of the most common surgeries worldwide [1]. In the United States alone, there are over 1 million total hip and total knee replacement surgeries performed each year [2]. The most common indication for hip and knee arthroplasty is symptomatic end-stage osteoarthritis [3,4]. Standard materials utilized for hip and knee implants include metals, ceramics, and polymers, in various combinations [5]. Some important properties that are considered when selecting these materials are friction, wear resistance, durability, and immunoreactivity of wear particles and other byproducts. Age is also an important factor when selecting implant types, as activity levels and biological characteristics change with aging [5,6]. The main causes of implant failures in arthroplasties are associated with aseptic loosening and periprosthetic joint infection.

Ten years ago in the US alone, there were over 40,000 total hip arthroplasties (THA) that underwent revision for aseptic loosening and it is expected that rates of revision will increase by 137% by 2030 [7]. Approximately 10% of implants fail within the first 10–20 years and 18% of these implant failures are due to aseptic loosening [8]. As the number of arthroplasties performed increases and is expected to double by 2050 [1], the number of arthroplasty revision surgeries performed for aseptic loosening will also increase. There have not been any studies that link aging itself with increased risk of aseptic loosening as it relates to arthroplasty. However, some clinical evidence has demonstrated that older age and osteoporosis are independent risk factors for poor integration of the prosthesis with bone which can result in implant loosening [9,10]. To reduce aseptic loosening, we focus on methods to improve initial implant integration with bone, known as osseointegration (OI).

The properties of the bone–implant interface are highly important in determining the longevity of joint replacements. Extensive evidence suggests that the properties of the implant itself, such as biomaterial choice, implant stiffness, and surface topography, play a key role in OI and bone remodeling following surgery [5,11,12,13,14]. The implant affects the surrounding mesenchymal stem cells (MSCs), which are essential progenitor cells for osteogenesis at the bone–implant interface [15]. The response of MSCs to an implant is affected by aging. Osteogenic differentiation of MSCs at the bone–implant interface is mechanosensitive, i.e., affected by mechanical stimulation at the bone–implant interface [14]. Aging is associated with differences in bone strains, loading, and motion during everyday activities like walking [16], which, in turn, may alter the response and stimulation of MSCs at the bone–implant interface.

Systemic factors, such as medications, systemic illness, and macrophage activity also affect stability at the bone–implant interface and are affected by aging. Research suggests that statins, for example, can help improve OI and bone density at the bone–implant interface [17]. Systemic illnesses that become more common with aging, such as osteoporosis and chronic kidney disease, may be associated with delayed OI and osteogenesis following joint replacement [15]. Macrophages, which are thought to play a key role in OI by regulating inflammation and immune reactions in response to new implants [15,18,19], may become dysregulated or deficient with aging [20,21]. Taken together, all of these factors associated with aging affect the bone–implant interface, OI and implant longevity.

In this review, we will discuss multiple strategies to maximize OI both mechanically via implant design and biologically. Current concepts such as 3D printing of implants and specialized implant coatings will be discussed in Section 4. Future directions include immunotherapeutics, biologic coatings, and genetic modification of MSCs. Currently there are multiple studies that focus on specific techniques for OI; however, there are relatively few review articles devoted to this topic, especially as it relates to aging. Our aim is to review the biological underpinnings of OI, examine how these processes are affected by aging, and discuss current and future strategies for optimizing OI. As the rate of joint replacements in the US and worldwide continues to increase, our understanding of and ability to maximize osteointegrative strategies will drive the success of joint replacement surgery for decades to come. Our understanding of how age affects OI is both relevant, timely, and important because arthroplasty is predominantly performed in an aging population.

## 2. General Cells for Osseointegration of Implants

Bone is a dynamic organ that supports skeletal muscles, regulates mineral homeostasis, and protects many organs in the body. Bone tissue is constantly renewed through bone remodeling processes. Bone regeneration after trauma, tumor resection, hip or joint replacement can be challenging in the aged population [22,23]. Homeostasis and metabolism of bone are maintained by the balance among bone-constituting cells, which include osteoclasts, osteoblasts, and osteocytes. These cells comprise the basic multicellular unit (BMU), a transient anatomic structure for insulating bone remodeling [24,25].

Osteocytes are known as the most mature differentiation state of osteoblasts; osteocytes permeate the interior of the bone and occupy lacunae. Each lacuna is located between the lamellae of the matrix and consists of one osteocyte. Osteocytes are derived from osteoblasts and are the most prevalent and advanced bone cells [26,27]. The osteocytes sense mechanical loading and produce soluble factors including sclerostin, dentin matrix protein 1 (DMP1), and osteoprotegerin (OPG) that regulate bone formation and resorption [28,29,30].

Osteoblasts and osteoclasts are present on bone surfaces. Generally, osteoblasts originate from mesenchymal stem cells (MSCs) and are responsible for bone formation (modeling) and replacing bone removed by osteoclasts (remodeling) [24]. Osteoblasts generate an extracellular matrix of bone. They secrete growth factors via matrix vesicles to lay down the osteoid structure. These growth factors include osteocalcin and alkaline phosphatase [31,32,33]. Osteoclasts are large, multinucleated phagocytic cells that originate from macrophage/monocyte cell lineages and specialize in bone resorption. Osteoclast precursors (OCPs) are attracted to specific skeletal sites and fuse to form the multinucleated osteoclasts that dissolve bone under regulatory factors such as macrophage colony-stimulating factor (M-CSF) and receptor activator of nuclear factor κB ligand (RANKL) [34]. Besides contributing to bone resorption, osteoclasts also participate in the long-term maintenance of blood-calcium homeostasis [35]. All three cell types (osteocytes, osteoblasts, and osteoclasts) are of importance in clinical practice with regards to the therapeutic effects on bone formation and remodeling.

In the event of bone regeneration after traumatic injury or a surgical intervention, the process of bone healing begins with the inflammatory phase. The immune system regulates bone cells, and bone regeneration responds to non-specific immune responses under injury conditions [36]. In the bone marrow, the immune system maintains hematopoietic stem cells (HSCs). Immune cells, such as T cells, B cells, neutrophils, and macrophages regulate bone integrity via the production of cytokines and chemokines [36]. For instance, activated T cells have been attributed to bone loss and increased fracture risk in chronic inflammatory disorders [37,38]. Furthermore, RANKL is not only an essential factor for osteoclastogenesis but is also associated with T cell activities [39,40]. Expression of RANKL on T cells allows them to interact with lymphoid tissue in the thymus and is essential to the tuning and calibration of the immune response [40]. Peripherally, T cells encounter antigen presenting cells (APCs) that are constantly presenting samples of the local environment. Under inflammatory conditions, T cells are providing RANKL to the APCs to prolong their interaction and enhance the inflammatory response [40]. T cells are important mediators of bone resorption [41]. B cell differentiation and maturation are closely related to bone cells as well. Activated B cells play an important role in inflammation-related bone diseases by directly regulating the expression of RANKL and activation of runt-related transcription factor 2 (RUNX2) [42,43]. Overall, both T cells and B cells play a role in bone homeostasis and are key participants in inflammation as it relates to aging and bone health.

Abnormal balance between bone formation and bone resorption results in diseases such as osteoporosis and osteopetrosis. Bone remodeling in BMU is a dynamic process as we have discussed above. In response to changes in the external environment [44], osteocytes initiate the process by recruiting osteoclast precursors followed by differentiation into mature osteoclasts. Then, the mature osteoclasts resorb excessive or damaged bone by secreting acid and proteolytic enzymes (cathepsin K (CTSK)) that dissolve collagen and degrade bone [45]. Cytokines released from the resorbed bone matrix, such as transforming growth factor beta (TGF-β) and insulin-like growth factor 1 (IGF-1) also affect osteoblast differentiation and osteoclast activity [46]. The release of IGF-1 was found to promote bone growth and prevent fractures [47]. Active TGF-β1 released from the bone matrix recruits mesenchymal lineage cells to resorptive surfaces and differentiates these cells into bone-forming osteoblasts [48]. This is followed by a reversal phase in which osteoclasts diminish, and osteoblast progenitors are recruited. Finally, mature osteoblasts produce extracellular proteins, including osteocalcin, alkaline phosphatase, and type I collagen to form the unmineralized osteoid and are subsequently mineralized through the accumulation of calcium phosphate [49]. To maintain bone homeostasis, the process is regulated through crosstalk among osteocytes, osteoclasts, and osteoblasts, by means of direct cell-cell contact and secretion of paracrine factors. These paracrine mediators include RANKL, Yes-associated protein (YAP), and Wnt family member 16 (WNT16), etc. [50,51,52,53]. Osteoblasts affect osteoclast formation, differentiation, or apoptosis through several pathways, such as OPG/RANKL/RANK, Wnt signaling, Fas/FasL pathways, and Ephrin2/ephB4 [54,55]. Conversely, osteoclasts influence osteoblastic activity and bone formation via the d2 isoform of vacuolar (H+) ATPase (v-ATPase) V0 domain (Atp6v0d2), complement component 3a, and semaphorin 4D.

Macrophages are key cells in the innate immune system and can be differentiated into osteoclasts in the presence of M-CSF and RANK [56]. Upon bone injury, damaged tissues release damage-associated molecular patterns (DAMPs) [57,58] to stimulate and polarize macrophages to a pro-inflammatory phenotype and produce inflammatory cytokines including the interleukin-1 family (IL-1), interleukin-6 (IL-6), and tumor necrosis factor alpha (TNF-α). The presence of these factors promotes RANKL-induced osteoclastogenesis. Macrophages also possess the capacity to promote osteogenesis. Macrophages with the anti-inflammatory phenotype enhance bone regeneration even under inflammation conditions [59,60]. Altogether, immune cells not only participate in bone homeostasis but also play important roles in regulating bone regeneration after a fracture or surgical intervention.

## 3. The Effects of Aging on Osseointegration and Bone Deposition at Bone–Implant Interfaces

### 3.1. Effects of Aging on the Bone Micro-Environment

Arthroplasty is primarily performed in an aging population, and the success of the surgery depends on bone deposition and ingrowth on an implant surface. During arthroplasty surgery, cuts in the bone are made and the orthopedic implant is placed. This is inherently traumatic for the bone micro-environment. We will discuss how the qualities of the bony microenvironment and the qualities of orthopedic implants are important for OI and how these qualities are affected by aging.

The cells at the bone–implant interfaces (osteoclasts, osteoblasts, osteocytes, as well as osteogenic progenitor cells) are involved in the healing process after arthroplasty. In addition, multiple growth factors are also present at the bone implant interface such as bone morphogenic protein (BMP), transforming growth factor-beta (TGF-β), fibroblast growth factor (FGF), insulin-like growth factor (IGF), vascular growth factor (VEGF), platelet derived growth factor (PDGF), and stromal derived growth factor (SDF1) [61]. Bone regeneration in the aged individual is complex and influenced by multiple factors, including systemic and local signaling molecules, osteogenic and resorptive cells, immune cells, and blood microcirculation [62]. Several factors such as hormonal change, oxidative stress-induced DNA damage, and poor diet contribute to tissue abnormality and imbalance in senescent bone homeostasis [63]. Via these mechanisms, the balance of bone homeostasis is disrupted with aging and the final result is osteoporosis. One of the most widely known examples of aging affecting hormonal change and bone health is the loss of estrogen as a key driver of bone loss mainly in women [64,65].

Oxidative stress-induced DNA damage is exacerbated with age and also as a negative effect on bone health. Increased oxidative stress and high levels of reactive oxygen species (ROS) have been known as inducers of senescence in bone cells, as evidenced by poor growth of cells in high oxygen concentrations [66]. While low levels of ROS can maintain bone homeostasis and a balance between osteoblasts and osteoclasts [67], excessive levels of ROS have been shown to cause cell death in osteoblasts and osteocytes and a reduction in bone architecture [68].

The metabolic rate slows with age and metabolic abnormalities are key hallmarks of aging. Dietary restriction is testified to extend the lifespan of an organism, thus affecting longevity and good health in humans [69]. Recent preclinical results have highlighted a decrease in nicotinamide adenine dinucleotide (NAD) metabolism with aging and its disruption of the Sirt1/FoxO/β-catenin pathway in osteoblast progenitors [70]. These studies showed that restoration of NAD levels in older animals could extend lifespan and promote good health [71].

Because of a variety of factors, including hormonal change, oxidative-stress-induced DNA damage, and slower rate of metabolism, bone mass declines significantly with age and the components of bone become more fragile.

### 3.2. Effects of Aging on Osteogenesis Capacity

At the cellular level, aging is associated with a concomitant reduction in bone formation and an increase in marrow adipogenesis [72]. Cells of mesenchymal origin regulate the bone formation process including bone marrow stem cells which are the progenitors to osteoblasts. They are supported by the multifaceted osteocytes, the most abundant cell type with an extensive canalicular network. The rate of bone formation depends on the level of availability of mesenchymal stromal cells (MSCs), progenitor pools, and sufficient recruitment of MSCs from those pools as well as the activity of individual osteoblasts on bone surfaces [73].

Age-related functional decline in osteoblasts due to increased apoptosis [74], decreased proliferation, impaired osteoblast differentiation [75], increased osteoblast senescence [76], and dysfunctional osteoprogenitors [77] leads to more marrow adipogenesis [78]. Aging-related decreases in the number and proliferative capacity of MSCs in the bone marrow has been observed in rats [79] and humans [80,81]. Human MSCs (hMSCs) from elderly individuals have lower proliferative and osteogenic potential than hMSCs from younger patients [82]. Additionally, the total number of nucleated cells in bone marrow aspirate decreases with age, regardless of sex [83]. Histologically measured mean wall thickness, a measure of the work performed by osteoblasts in the BMU, declines with advancing chronological age in both sexes [84]. In mice, defective bone formation with aging is also due to a reduction in MSC progenitor pools, defective activation or differentiation of these progenitors, or alterations in allocation towards the adipogenic lineage [85]. Taken together, the net result is decreased bone formation and increased bone marrow adipose tissue (BMAT).

Previous studies suggested that telomere length might contribute to impaired osteogenesis in aged MSCs. Osteoblast differentiation is inhibited in a mouse model of Werner’s syndrome (premature aging) with shortened telomere length [86]. Further, hMSCs from the elderly have significantly shorter mean telomere restriction fragments, which may contribute to the difficulty of hMSCs to undergo osteogenic differentiation [87].

### 3.3. Effects of Aging on Osteoclasts

Osteoclasts, large multinucleate cells that have a hematopoietic origin, are responsible for resorbing the bone matrix and are regulated by extracellular signals secreted by the osteoblasts and osteocytes, while being supported by progenitors such as bone marrow monocytes or macrophage precursors [88,89,90]. Differentiation of osteoclasts requires binding of RANKL to the RANK receptor on the osteoclast surface [91,92] together with the secretion of macrophage colony-stimulating factor (M-CSF) by osteoblasts and bone marrow stromal cells [93]. In the process of osteoclastogenesis, bone-marrow-derived macrophages differentiate into tartrate-resistant acid phosphatase (TRAP)-positive preosteoclasts under the action of the RANKL receptor activator [94]. Mononuclear preosteoclast cells are then combined with each other to form multinucleated mature osteoclasts.

The mechanisms that cause bone loss via aging are not completely clarified, but the number of osteoclasts increased with aging, which led to increased bone resorption, and resulting in osteoporosis [72]. Previous studies reported that the increase in osteoclast number with aging is associated with several factors such as RANKL and oxidative stress [72,95]. Osteoprotegerin (OPG) is secreted by a variety of cells including osteoblasts and OPG reduces bone loss by blocking the combination of RANKL and RANK [96]. The OPG levels are reduced due to the aging-related decrease of osteoblasts, thereby activating osteoclast resorption and causing osteoporosis [63]. Sirtuin 3 (SIRT3) is an important protein in mitochondrial metabolism, and SIRT3 could facilitate osteoclastogenesis and bone loss by activating the mTOR pathway in aging transgenic overexpressing SIRT3 mice [97]. Loss of SIRT3 impaired bone resorption by reducing mitochondrial respiration and mitophagy of osteoclasts but did not affect the osteoclast number [98]. These findings indicate that elevated SIRT3 may promote bone loss in aged cells, partially due to an imbalance in bone resorption.

### 3.4. Effects of Aging on Healing Potential

The capacity of bone healing is hindered with age. Bone healing relies on recruitment of MSCs followed by formation of extracellular matrix, angiogenesis, and revascularization. These processes are impaired in osteoporotic bone via changes in cell behavior, vascular response, and extracellular matrix activity [99,100,101]. For example, post-menopausal, estrogen-deficient mice have a decreased numbers of MSCs and decreased levels of angiogenesis [99]. Other studies in murine models have demonstrated age-dependent delays in osteoblast differentiation and cell proliferation [99]. Hypoxia Inducible Factor-1-alpha (HIF-alpha) and VEGF expression is suppressed in aged murine models, which impairs vascularization required for new bone growth [100]. Furthermore, age-related changes in bone matrix composition also delay healing [101]. The combination of delayed MSC recruitment, suppressed angiogenesis, and sub-optimal bone matrix composition makes healing around a metal implant a challenge in the aged individual.

MSCs and macrophages are critical for bone healing and are both affected by age. After arthroplasty surgery, MSCs migrate and attach to the surface of new implants as soon as one day after surgery [102,103]. MSCs are involved in osteogenesis and bone remodeling around the surface of the new implant [102]. OI is also an immune-mediated process; macrophages play a key role in promoting bone formation around new implants [15]. Mouse models suggest that age-related changes in macrophages are associated with dysregulated macrophage polarization and delayed fracture healing [104].

OI by MSCs, macrophages, and other cells is heavily dependent on the properties of the implant, including material, shape, topography, size, and wear particles [102]. Additionally, OI by MSCs and macrophages is believed to be sensitive to mechanical stimuli, such as loading and activity levels of the host [105]. Aging is linked to changes in bone strains, loading, and movement patterns during daily activities like walking [16], which could potentially affect the OI potential of MSCs and macrophages at the bone–implant interface as they respond to and are stimulated by mechanosensitive stress.

Interestingly, there is no evidence to support a higher rate of arthroplasty implant failure (long or short term) in elderly patients. Most studies that have looked at the relationship between aging and implant failure come from the field of oral and maxillofacial surgery. One study investigated the OI of oral implants in older and younger adults and found no difference in implant survival between groups [106]. There was 100% implant success in both groups. Another study looked at implant loss in elderly (>65 years old) compared to younger (<55 years old) patients and found a similarly low rate of early implant loss in both groups. However, there may have been a slight tendency for a higher early implant loss (EIL) rate in patients > 80 years old [107]. From currently published research, age itself does not seem to compromise OI and, if at all, then only slightly and at a later stage of life. However, age-related comorbidities like chronic inflammation, decreased activity, and osteoporosis may contribute to poorer OI and are associated with other adverse effects like mechanical stability of implant and biological response of bone [108].

## 4. Strategies to Improve Osseointegration

Despite 40 years of advances in implant design, biomechanical failures are still a problem. Aseptic loosening is one of the main reasons why joint replacements fail. The main reason for failure of total hip arthroplasty is aseptic loosening with particle-induced periprosthetic osteolysis followed by infection [109]. One study found that 25% of active patients (UCLA activity scale 8–10) developed femoral osteolysis five to 10 years postoperatively [110]. While the active group of patients had higher postoperative satisfaction in general, there were questions about whether early and higher strain and loads across the implant may have contributed to the higher rate of loosening. Yamada et al. reports up to 70% of total joint arthroplasties require a replacement surgery within 10 years as a result of periprosthetic osteolysis [111].

There are a multitude of strategies to improve OI and we will discuss a variety of factors here including mechanical considerations (implant design, topography, and porosity) and biological considerations (such as implant coatings, immune modulation, osteoinductive biologic therapies). Anti-microbial therapies are essential for preventing infection and allowing OI to occur at the bone–implant interface.

### 4.1. Improving Osseointegration through Engineering and Implant Design

The implant material is important for mechanical strength. Titanium alloy (Ti6Al4V) is primarily used for orthopedic implants because of its mechanical strength and relatively lighter weight compared to stainless steel. Despite this, titanium alloy still has a relatively higher elastic modulus compared with bone tissue. This contributes to a phenomena known as “stress shielding” whereby there is a reduction in bone density as a result of removal of the typical stress from the bone by an implant. The stress-shielding effect can create poor bone integration with scaffolds and eventually can lead to aseptic loosening [112]. The ideal implant has the lightweight properties of titanium alloy and a similar elastic modulus as bone without sacrificing mechanical strength.

Micromotion refers to the motion of the implant relative to the bone during loading. Micromotion at the bone=implant interface can prevent adequate healing, leading to osteolysis and aseptic loosening. Motion at the bone–implant interface can also generate metal wear particles that incite an inflammatory reaction. The body seeks to eliminate these foreign molecules, but the metal wear particles cannot be easily phagocytosed by macrophages [113]. Particle-stimulated macrophages release inflammatory cytokines and chemokines and their persistence contributes to local chronic inflammation [114]. However, micromotion by itself, (without the generation of wear particles) also decreases osteointegration and stability [115,116,117]. One solution to this problem is gap-bridging coatings, designed to eliminate any microscopic space between the implant and bone surface [118]. Implant coatings made from hydrogel, foam, or deformable metal can expand and eliminate microscopic gaps at the bone–implant interface, but these techniques are not widely used [119].

The design of the orthopedic implant is essential to reduce micromotion, promote OI, and in some cases, prevent infection. The 3D micro and nano topography of the implant are important for bony ingrowth [120]. Engineered surface implants are porous at the micro- and nano- level to mimic the environment of bony trabeculae and increase surface area. Surface modifications are a valuable way that we can influence bony ingrowth. Techniques such as grit blasting or plasma sprayed coatings are utilized to design the implant surface topography at the nanoscale to promote OI [121,122]. A study published recently evaluated 235,500 cemented and 10,749 uncemented primary total knee arthroplasties and showed that, when broken down by surface modification of the implant (cemented, porous hydroxyapatite (HA), porous uncoated, and grit-blasted uncoated), the porous HA and porous uncoated surfaces performed the best in terms of revision rates for aseptic loosening, and grit-blasted implants underperformed [123].

3D printing is being utilized in arthroplasty implant design. There are “off the shelf” components as well as custom 3D printed implants. Custom implant design utilizes preoperative radiographs or CT imaging to match native anatomy and reduce stress shielding [122]. However, custom one-of-a-kind implants are mainly used in complex acetabular revisions and are not mainstream, likely due to the labor and cost involved in their manufacturing [124]. However, in patients with poor bone stock, bony defects, anatomic variances (due to dysplasia, trauma, or revisions), or for the purposes of pre-operative planning and decision making, 3D-printed models have the potential to be very useful. In some cases, implants can be 3D printed using “trabecular titanium”, as described by Regis et al. [125]. Surface nanoporosity was shown to have a direct impact on cell adhesion to titanium implants [14,126] and the size of the pores has an impact on cell adhesion. As pore size increases, the proliferation of integrin that seeds onto the scaffold increases and reaches a maximum value at a pore size of 600 μm and then subsequently decreases [112]. When assessing the applicability of 3D printing in orthopedic implants based on qualitative evidence, it appears there is insufficient evidence to determine whether the advantages can be offset by cost and availability.

MSCs at the bone–implant interface have mechanoreceptors and respond to physical or structural changes in their environment. Metal implants have been shown to stimulate osteogenic differentiation of MSCs [126]. However, there is still no agreed upon optimal surface pattern for inducing osteogenic differentiation of MSCs, and the majority of these studies have been conducted in vitro. These examples demonstrate that the mechanics of the implant have an impact on the biological response of the surrounding cells.

### 4.2. Improving Osseointegration through Biological Modifications

#### 4.2.1. Attracting Chemokines to Stimulate Bone Growth

New biological approaches to enhancing OI are under development. Mesenchymal stem cells (MSCs) stimulate bone growth and healing and are present in the periosteum and endosteum of the bone [127]. Additional MSCs are recruited by chemokines like SDF-1 and CXCR4 [128]. It is critical that chemokines are present at the implant surface in order to attract more MSCs to the site and stimulate osteogenesis. A novel way of enhancing the number of chemokines at the implant surface is the use of exosomes that can deliver signaling molecules to the bone–implant surface. Exosomes are small (30–120 nm size) membrane-bound vesicles that can carry cytokines, chemokines, growth factors, and signaling molecules. In the past, exosomes have been used to deliver MiR-126 (a chemokine) to increase angiogenesis in endothelial cells [129]. In a rat model of femur fractures, MSC-derived exosomes enhanced bone healing and angiogenesis [130]. Clinical trials have demonstrated the feasibility of extracellular vesicle immunotherapy in humans; however, broader application of this technology is limited by the ability to efficiently load drugs into the exosome and methods of controlling biodistribution [118].

#### 4.2.2. Reducing Chronic Inflammation

We can directly target and promote OI biologically, but we can also indirectly achieve this aim by reducing chronic inflammation. Macrophages are key cells in bone formation and are also modulators for acute and chronic inflammation. They secrete cytokines and chemokines (IL-1, IL-6, and TNF-alpha), as well as growth factors (TGF-B, BMP, and PDGF) during the acute inflammation stage. Broadly speaking, there are two subtypes of macrophages: M1 and M2. M1 macrophages are pro-inflammatory and promote bone resorption. M2 macrophages are anti-inflammatory and promote bone formation [131]. During bone healing, macrophages begin as the M1 phenotype (proinflammatory). Appropriate resolution of the inflammatory phase depends on M1 polarizing to M2 macrophages, which are more closely involved in tissue regeneration [118,132,133].

Acute inflammation is essential to initiate bone healing after arthroplasty surgery. Acute inflammation stimulates angiogenesis, promotes MSC differentiation into osteoblasts, and the transition of M1 macrophages to M2 macrophages [134]. This process lasts about 1 week in humans [118,135,136]. Then, it is crucial that inflammation subsides. Inflammation that does not subside and becomes chronic, results in impaired osteogenesis [118,137]. As we age, there is a progressive failure to polarize macrophages from an M1 to an M2 phenotype, resulting in a persistent M1 pro-inflammatory state which causes increased bone resorption and decreased bone formation [118,138]. Theoretically, this would lead to higher rates of aseptic loosening with advanced age; however no studies to date have investigated the relationship between the rates of aseptic loosening and aging.

Advanced age correlates with increased levels of inflammation at the cellular level which has a negative effect on bone healing. One approach to improving osteosynthesis is to prevent the cycle of chronic inflammation. Other studies have investigated genetically modified MSCs that secrete cytokines, which can curb the chronic inflammation that occurs with aging. For example, lentivirus-transduced IL-4 over-expressing MSCs facilitated bone healing in murine models with long bone critical size defects by inducing polarization from M1 (pro-inflammatory) to M2 (anti-inflammatory) macrophage phenotypes [118,139]. Another example of this is NF-kB sensing-IL-4 secreting MSCs. The elevated NF-kB during chronic inflammation triggers these cells to secrete IL-4 (anti-inflammatory cytokine). In a study by Antoci et al., local injections of NF-kB-IL4-MSC suppressed chronic inflammatory osteolysis by increasing the M2/M1 macrophage ratio [138,140,141]. A study by Pan et al. [142] reported that lithium containing bioceramics can inhibit pro-inflammatory cytokines. They demonstrated reduced calvarial osteolysis in murine models via micro-CT and histological analysis. Taken together, genetically modified MSCs can alter the M1 to M2 macrophage ratio and, therefore, moderate chronic inflammation that can impair bone healing.

#### 4.2.3. Biological Modifications to Implant Coatings

The most common implant coating that is currently in use is the hydroxyapatite (HA) coating; however data their ability to improve OI has been inconsistent. When comparing HA-coated vs. non-HA-coated stems there, was no significant difference in implant survival [143]. However other studies have reported advantages of HA-coated implants compared to porous implants [144]. A meta-analysis looking at twelve RCTs and nine observational studies showed that HA-coated implants improved the Harris hip score, reduced the incidence of thigh pain and incidence of femoral osteolysis, but had no advantages in implant survival [145]. There remains potential for implant coatings to stimulate osteogenesis and prevent infection. Recently, a BMP-2-loaded composite was approved by the FDA. When combined with HA, beta-tricalcium phosphate microsphere/poloxamer 407-based hydrogel recombinant human (rh) BMP-2 demonstrated enhanced differentiation of osteoblasts and increased volume and quantity of newly formed bone in a rat model [146]. Implants can also be coated with calcium phosphates (such as HA) to promote bony ingrowth via increased osteoblast proliferation and alkaline phosphatase production [147]. HA-coated implants have been shown to have increased torque strength, pullout strength, and bone ingrowth [145]. Coating titanium implants with chitosan (biopolymer) also increased osteoblast adhesion to the implant [148].

#### 4.2.4. Preventing Infection

Acute microbial infection at the implant site can prevent OI. With age and osteoporosis, the risk of infection is higher, likely because the body’s immune response is compromised [149]. Because a majority of patients undergoing arthroplasty surgery have osteoporotic bone [150], preventing infection is of high importance to ensure bone healing around the implant. Research dedicated to fracture-related infection showed that osteoporotic bone had more severe bone lysis, higher bacterial load, and longer delays in fracture healing [151]. The authors also found that healing in osteoporotic bone showed poorer callus remodeling, more severe periosteal reaction, and more severe bone lysis [151]. There was also more extensive cortical bone microbial colonization in osteoporotic bone, which was more resistant to systemic antibiotics; this represents another cause of impaired bone healing.

Preventing infection is challenging because bacteria preferentially adhere to the implant and produce an extracellular biofilm which protects them and makes them significantly more challenging to eradicate [152,153,154]. To decrease acute microbial adhesion, patients undergoing total joint arthroplasty procedures are routinely given perioperative antibiotics. Additionally, surgeon preference dictates whether antibiotics are applied directly into the joint at the time of surgery. In cemented arthroplasties, the polymethylmethacrylate (PMMA) cement is pressurized and often has antibiotics mixed in to improve fixation, reduce gaps, and prevent infection, respectively. These techniques are aimed at reducing infection in the short-term. However, orthopedic implants are designed to last many years [155]. Preventing infection in the long term remains a challenge. Heavy metal-based surface modifications of titanium alloy prosthesis have shown effectiveness in reducing PJI [156]. Newer studies have shown the implants coated in silver ions are bactericidal and that the ions are slowly released [157]. An in vivo rodent study involving femoral implants showed no bacterial activity at 8 weeks with a silver releasing implant [157]. This is promising, but 8 weeks is still relatively short-term compared to the expected lifetime of an orthopedic implant, and longer-term studies are needed.

#### 4.2.5. Osteoinductive Techniques to Maximize Osseointegration

So far, we have reviewed various techniques for osteoconduction, or the promotion of bone growth via mechanical design and implant coatings. Osteoinduction refers to the processes by which one tissue causes a second undifferentiated tissue to differentiate into bone. It can be described in three distinct phases: (1) Mesenchymal cell recruitment; (2) Mesenchymal differentiation into bone-forming osteoblasts; (3) Ectopic bone formation in vivo, or when implanted into extra skeletal locations [158]. BMP-2 has been used as an osteoinductive coating for orthopedic implants and is capable of enhancing MSC chemotaxis [158]. Strong results are evidenced by significantly increased bone formation and density when BMP-2 was applied to tibial bone defects in sheep [159,160]. The challenge is recruitment of osteogenic cells to the location where new bone growth is desired. Kitaori et al. [128] demonstrated recruitment of osteogenic cells through implants that release signaling factors, such as stromal-cell-derived factor 1 (SDF-1). This chemokine can mobilize MSCs to the site of the implant through their CXCR4 surface receptor and results have been promising with in vitro and in vivo mouse models demonstrating that BMSCs migrated in a dose-dependent manner to the chemical gradient of SDF-1. Furthermore, inhibitions of SDF-1 reduced new bone formation [128]. Future studies are needed to investigate how these processes are affected by aging.

## 5. Future Directions

### 5.1. Next Steps in Engineering and Implant Design

Implants can be coated with various materials to both promote bone growth and prevent infection as we have discussed. Most coatings aim to do one or the other. A clear next step is to develop coatings that have both osteoconductive and antimicrobial properties. The work is ongoing—a recent study showed that coatings combining calcium phosphate with antimicrobial peptides demonstrated enhanced bone–implant contact in a rabbit model [161]. The next step is to approve this technology for use in human subjects. Expandible implant coatings (like titanium braded wire constructs or hydrogels) have been utilized in rodent models but have yet to undergo human trials.

With increasing access to 3D printing, there is ample opportunity for growth in orthopedic implant design. Due to the increasing rates of arthroplasty worldwide, orthopedics face the challenge of keeping up with demand. This hinges on the cost of material and the speed of manufacturing [162]. In a study by Dall’Ava et al. [163], 3D-printed titanium alloy implants were compared to conventional implants. They found that that 3D printed implants were significantly more porous than conventional implants. They found that 3D printing enabled the fabrication of more complex porous structures that maximize bone integration. Other researchers have demonstrated the potential of 3D printing to create reservoirs for implants to elute antibiotics [164]. Whether 3D printing of orthopedic implants is more cost effective and can be produced efficiently on a large scale remains to be seen.

Titanium surfaces loaded with antibiotics offer an exciting step forward. As Esteban et al. describes, this can be accomplished via nanostructured surfaces or covalently bound antibiotics [156]. Nanotubes can be manufactured via hydrothermal synthesis or anodization and can be loaded with antibiotics like vancomycin or gentamycin. Alternatively, antibiotics can be covalently bound to titanium surfaces. For example, covalently binding vancomycin to titanium implants has been performed in preclinical murine models [156]. There are no clinical trials yet in humans, mainly because of the cost of clinical trials that would be needed to demonstrate efficacy in humans. Funding and collaboration on a large scale, including pharmaceutical companies, regulatory agencies, and health care leaders, is likely needed.

### 5.2. Next Steps in Biological Strategies to Improve OI

From a biological perspective, a potential future direction in OI is the use of exosomes to deliver signaling molecules to the bone implant surface. Extracellular vesicles have historically carried different molecules including RNA. Considerable work has been dedicated in recent decades to macromolecule transport and accurately engaging extracellular targets; however, success has been limited [130]. From an immunologic standpoint autologous EVs are an attractive option; however, they are difficult and time intensive to isolate and scalability is a limiting factor.

Implant coatings can be designed to enhance MSC recruitment to the implant site in osteoporotic fractures. In a study by Chen et al., titanium implants coated with porous HA and phosphorylated osteogenic growth peptides were shown to improve osteogenic differentiation of MSCs. They also demonstrate that ROS-triggered release in the osteoporotic environment guides MSC migration to the implant site [165]. In this example, combining implant material and osteoinductive coatings is a promising step forward.

Another future direction for improving OI lies in immunomodulation. We previously discussed how lentivirus-transduced IL-4 over-expressing MSCs facilitated bone healing in murine models by inducing macrophage polarization from M1 to M2 and inhibiting chronic inflammation. In theory, chemokine overexpressing MSCs could be used to promote bone growth. For example, TGF-β1 (recruits mesenchymal lineage cells to resorptive surfaces and differentiates them into bone-forming osteoblasts) may be able to enhance bone healing at an implant interface if it could be over-expressed by local MSCs. Practically, delivering these genetically modified MSCs to the appropriate site would be a challenge in vivo. Further research is needed to evaluate the safety and efficacy of genetically-modified-MSC therapy in humans as current research has only been conducted in animal models. The FDA in the United States also presents a high bar for approval of any genetically modified treatment in human subjects.

One of the additional challenges with MSC therapies is the variability in manufacturing protocols. Almost every step in the process, from donor source, isolation procedures, harvesting, preservation, to dose delivery, is variable depending on the laboratory. Additionally, the composition of the culture medium has an impact on the MSC heterogeneity, clonogenicity, proliferative capacity, and surface immunophenotype [166]. The variability in manufacturing of MSCs and the lack of regulatory protocols presents a barrier that impedes the scalability of MSC therapies along the clinical drug pipeline [166].

Bioceramics such as calcium phosphate, HA, and calcium silicate (Ca-Si) are the future direction in implant coatings. Calcium-phosphate-like coatings, such as HA have been shown to improve OI of a cementless metallic prosthesis [167]. HA interacts well with the bone surface but has a mismatch in its expansile ability compared to titanium alloy which may cause failure under tensile loads [168]. Ca-Si based ceramics have a higher bonding strength with titanium alloy compared to HA [169]. They also support attachment, proliferation, and differentiation of osteoblasts because of the release of Ca^2+^ and Si^2+^ ions. The dose-dependent antibacterial activity of Ca-Si has also been demonstrated in early studies [168]. The Ca-Si based ceramics have comparable CTE with titanium alloy and, therefore, improved bonding strength. So far, the Ca-Si coatings have shown excellent in vitro bioactivity, with a rough microstructure and higher bonding strength compared to HA. Using plasma-spraying, a thinner layer can be achieved, which helps mimic the shear strength of cortical bone. Poor chemical stability (rapid degradation rate) are the barriers to long term durability and in vivo studies [169,170].

The biological environment around the implant is also affected by local targeted deliver of antibiotics via implant coatings [171]. Silver-coated implants continue to be at the forefront of arthroplasty implant for their antimicrobial properties and lower rates of PJI [172]. There is an opportunity to push the needle forward by adding growth factors to the coatings, thereby producing a coating with osteoconductive and osteoinductive properties [171].

Future directions in implant design to promote OI include the controlled delivery of biomolecules that enhance the natural wound healing response, increase bone formation, and modulate chronic inflammation.

## 6. Conclusions

OI is a complex biological process involving osteocytes, osteoblasts, and osteoclasts acting in homeostasis. The interactions between these cells, which results in bone formation or degradation, is mediated by paracrine signaling factors related to each cell type as well as the balance between acute and chronic inflammation that is necessary for bone turnover. Aging is associated with the impairments of these factors. Solutions to improve bony ingrowth and decrease aseptic loosening can be directed at maximizing concentrations of growth factors and chemokines, reducing chronic inflammation, and engineering implants that reduce micromotion, utilize 3D printing to maximize porosity, and are coated with osteoconductive and osteoinductive factors.

## Abbreviations

Osseointegration (OI), hydroxyapatite (HA), basic multicellular unit (BMU), mesenchymal stem cells (MSCs), dentin matrix protein 1 (DMP1), osteoprotegerin (OPG), osteoclast precursors (OCPs), macrophage colony-stimulating factor (M-CSF) and receptor activator of nuclear factor κB ligand (RANKL), hematopoietic stem cells (HSCs), antigen presenting cells (APCs), runt-related transcription factor 2 (RUNX2), cathepsin K (CTSK), transforming growth factor beta (TGF-β), insulin-like growth factor 1 (IGF-1), Yes-associated protein (YAP), and Wnt family member 16 (WNT16), release damage-associated molecular patterns (DAMPs), interleukin-1 (IL-1), interleukin-6 (IL-6), tumor necrosis factor alpha (TNF-α), bone morphogenic protein (BMP), transforming growth factor-beta (TGF-β), fibroblast growth factor (FGF), insulin-like growth factor (IGF), vascular growth factor (VEGF), platelet derived growth factor (PDGF), and stromal derived growth factor (SDF1), reactive oxygen species (ROS), nicotinamide adenine dinucleotide (NAD), human MSCs (hMSCs), bone marrow adipose tissue (BMAT), tartrate-resistant acid phosphatase (TRAP), Sirtuin 3 (SIRT3), hypoxia Inducible Factor-1-alpha (HIF-alpha), early implant loss (EIL), hydroxyapatite (HA), polymethylmethacrylate (PMMA), extracellular vesicles (EV), and calcium silicate (Ca-Si).
