# Correction: Pius et al. Effects of Aging on Osteosynthesis at Bone–Implant Interfaces. *Biomolecules* 2024, *14*, 52

**DOI:** 10.3390/biom14030340

**Published:** 2024-03-12

**Authors:** Alexa K. Pius, Masakazu Toya, Qi Gao, Max L. Lee, Yasemin Sude Ergul, Simon Kwoon-Ho Chow, Stuart Barry Goodman

**Affiliations:** 1 Department of Orthopaedic Surgery, School of Medicine, Stanford University, Stanford, CA 94063, USAyaseminsudeergul@gmail.com (Y.S.E.);; 2 Department of Bioengineering, Stanford University, Stanford, CA 94305, USA

Max L. Lee was not included as an author in the original publication [1]. The corrected Author Contributions statement appears here. The authors state that the scientific conclusions are unaffected. This correction was approved by the Academic Editor. The original publication has also been updated.
